# High-confidence cancer patient stratification through multiomics investigation of DNA repair disorders

**DOI:** 10.1038/s41419-022-05437-w

**Published:** 2022-11-26

**Authors:** Garik V. Mkrtchyan, Alexander Veviorskiy, Evgeny Izumchenko, Anastasia Shneyderman, Frank W. Pun, Ivan V. Ozerov, Alex Aliper, Alex Zhavoronkov, Morten Scheibye-Knudsen

**Affiliations:** 1 Center for Healthy Aging, Department of Cellular and Molecular Medicine, University of Copenhagen, Copenhagen, Denmark; 2 Insilico Medicine, Hong Kong, China; 3 Department of Medicine, Section of Hematology and Oncology, University of Chicago, Chicago, IL USA

**Keywords:** Tumour biomarkers, Cancer genetics, Prognostic markers

## Abstract

Multiple cancer types have limited targeted therapeutic options, in part due to incomplete understanding of the molecular processes underlying tumorigenesis and significant intra- and inter-tumor heterogeneity. Identification of novel molecular biomarkers stratifying cancer patients with different survival outcomes may provide new opportunities for target discovery and subsequent development of tailored therapies. Here, we applied the artificial intelligence-driven PandaOmics platform (https://pandaomics.com/) to explore gene expression changes in rare DNA repair-deficient disorders and identify novel cancer targets. Our analysis revealed that CEP135, a scaffolding protein associated with early centriole biogenesis, is commonly downregulated in DNA repair diseases with high cancer predisposition. Further screening of survival data in 33 cancers available at TCGA database identified sarcoma as a cancer type where lower survival was significantly associated with high CEP135 expression. Stratification of cancer patients based on CEP135 expression enabled us to examine therapeutic targets that could be used for the improvement of existing therapies against sarcoma. The latter was based on application of the PandaOmics target-ID algorithm coupled with in vitro studies that revealed polo-like kinase 1 (PLK1) as a potential therapeutic candidate in sarcoma patients with high CEP135 levels and poor survival. While further target validation is required, this study demonstrated the potential of in silico-based studies for a rapid biomarker discovery and target characterization.

## Introduction

Maintenance of genomic integrity has a pivotal role in preventing the development of age-associated diseases such as cancer and neurodegeneration. Exposure of somatic cells to multiple endogenous and exogenous stressors results in the accumulation of unrepaired DNA lesions and rearrangements, leading to overall genome instability, that is a hallmark of cellular transformation and cancer progression [1, 2]. Molecular mechanisms underlying this condition include alterations in the DNA repair machinery, replication stress, altered transcriptional responses and changes in cell cycle regulation [3–5]. Multiple types of solid and bone marrow malignancies display distinct defects in certain pathways of the DNA damage response (DDR), and several therapeutic strategies targeting repair mechanisms have been previously developed and validated in clinical settings [6, 7]. For example, higher levels of genome instability are seen in breast cancer cells carrying mutations in BRCA genes, which play a critical role in double-stranded breaks repair. BRCA-deficient cells with defective homologous recombination, rely on more error-prone non-homologous end joining repair and are sensitive to PARP inhibitors, providing a strategy for selectively inducing synthetic lethality in cancer cells [8]. Additionally, defects in DNA mismatch repair genes MLH1 and MSH2, associated with a subset of colorectal tumors with microsatellite instability, may lead to abundant mutation-derived neoantigens that trigger a robust immune response to checkpoint inhibitors therapy [9, 10]. Bone marrow-derived cancers are also characterized by mutations in key DNA damage response and DNA repair genes. For instance, mutations in ATM, a key gene for DDR activation, and TP53 have frequently been detected in several types of lymphomas [11, 12].

Importantly, several premature aging diseases caused by genetic impairments in DNA repair machinery are also associated with increased cancer risks [13, 14]. For example, inherited mutations in the ATM lead to ataxia-telangiectasia (A-T), a rare premature aging disease with features of neurodegeneration and increased risks of developing lymphomas and various solid malignancies, including breast and digestive tract cancers [14–17]. Another example includes Nijmegen Breakage syndrome (NBS), where mutations in NBS1 gene, a member of the MRE11-RAD50-NBS1 (MRN) complex serving as sensor of DNA damage, lead to immunodeficiency and higher risk of developing cancer [13, 18–22]. Furthermore, mutations in the RecQL DNA helicase WRN may lead to Werner syndrome, evidenced by increased incidence of cardiovascular diseases and cancer development, in particular sarcomas, skin and thyroid malignancies. While heritable diseases with impaired DNA repair function are characterized by a significant genetic and phenotypic variability between each other [13, 14], increased cancer risk is a clinical phenotype shared across multiple DNA repair disorders. Since not all patients with DNA repair disorders develop malignant diseases, identification of altered “cancer-prone” genes associated with tumorigenic processes could, therefore, lead to the discovery of novel cancer risk stratification biomarkers and subsequent therapeutic targets.

Identification of therapeutic targets is a crucial step of the drug discovery process. Erroneous targets selected at the early stage of drug development may result in a costly drug discovery program and failed clinical trials. While development of the automated approaches for drug target discovery is critical for maximizing the success rate, it still remains a challenging task due to a number of limitations, such as complexity of the data, batch effects and others. While these challenges cannot be resolved by the traditional methods, such as gene expression arrays, artificial intelligence (AI)-driven approaches have recently demonstrated their efficacy in this setting across multiple diseases including embryonic-fetal transition [23] and muscle aging [24]. Advanced pathway analysis and AI algorithms applied to multiomics data are capable of identifying novel targets and biomarkers even when the prior evidence is insufficient, especially when it comes to the most frequently available dynamic omics data including gene expression and proteomics [25–27] as well as not as abundant data types such as phosphorylome [28] and even microbiome [29]. Moreover, AI has also been successfully applied to already existing targets where crystal structures are not available [30].

**Fig. 1:**
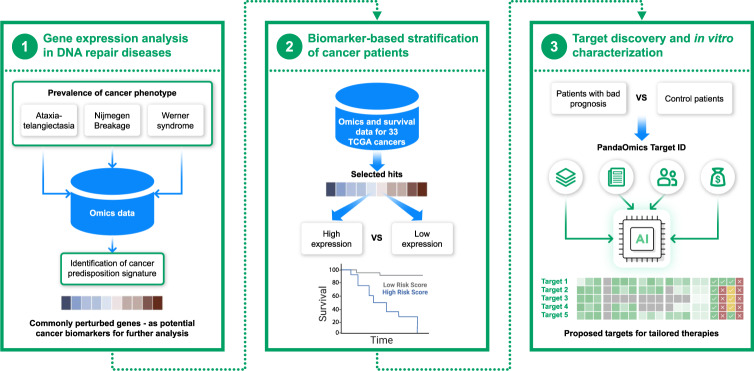
Schematic representation of the PandaOmics application for a rapid biomarker discovery and target characterization in cancer. Gene expression signatures have been examined in DNA repair diseases with high cancer predisposition (1), followed by the analysis of the most significantly perturbed genes as potential biomarkers stratifying cancer patients based on their survival rates (2). The group of patients with low survival outcomes have been further used for identification of potential therapeutic candidates for cancer treatment via PandaOmics Target ID approach (3). Data types used to generate target hypotheses included: omics-, text-, key opinion leaders (KOLs) and funding- based scores (see materials and methods section as well as the user manual (https://insilico.com/pandaomics/help).

In the current paper, we applied a three-tier approach where (1) knowledge about diseases with cancer prevalence enabled (2) identification of biomarker genes and (3) subsequent discovery of possible therapeutic targets (Fig. 1). We took advantage of the cancer-prone phenotype overlapping between diverse DNA repair diseases with discrete phenotypic prevalence (neurodegeneration, immunodeficiency and cardiovascular disease), and applied differential gene expression analysis driven by AI-based PandaOmics platform to identify those genes that are commonly perturbed among selected diseases and that could be associated with cancer progression. The most significantly dysregulated gene CEP135 was further discovered to be used as a novel biomarker that stratifies sarcoma patients with better and poor survival outcomes among the TCGA database of 33 various cancer types (Fig. 1). Furthermore, using PandaOmics-based TargetID we revealed gene candidates that could be used as targets for drug discovery for more efficient elimination of cancer cells in sarcoma patients with high expression of CEP135 and lower survival probability.

## Results

### Phenotype-based clusterization identified DNA repair diseases with high cancer predisposition

**Fig. 2:**
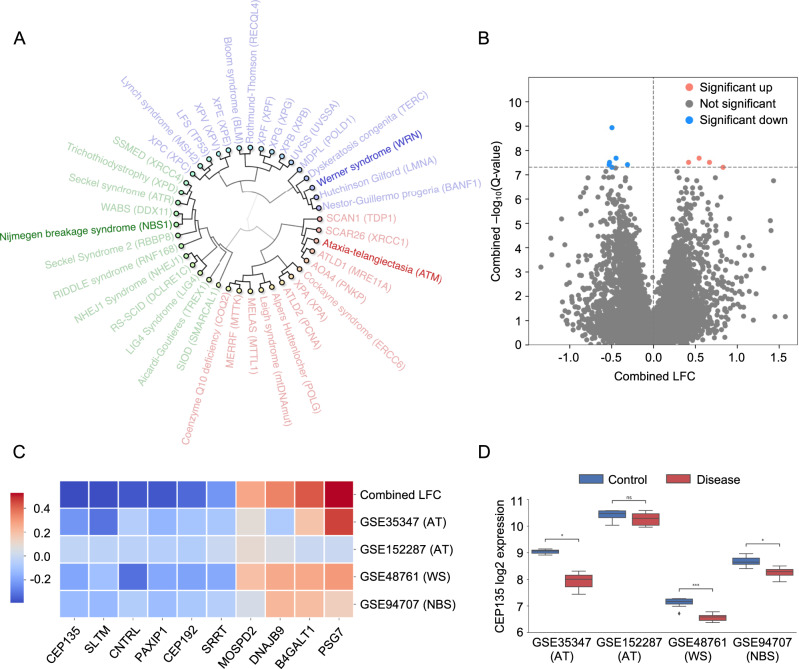
Identification of common gene expression signatures among DNA repair diseases with cancer-prone phenotype. **A** Hierarchical clustering of DNA repair diseases with selected three diseases for gene expression analysis (Ataxia-Telangiectasia, Nijmegen Breakage syndrome and Werner syndrome). **B**, **C** Visualization of top 10 perturbed genes among three diseases on a volcano plot and heatmap (LFC—log-fold-changes). **D** Gene expression changes of CEP135 gene between disease and control samples for all analyzed datasets (ns—not significant, *p-value < 0.05, ***p-value < 0.001, two-tailed Mann–Whitney test).

In order to select diseases for subsequent gene expression analysis and identification of the novel cancer biomarkers, we performed hierarchical clustering based on the analysis of common clinical phenotypes that are prevalent in multiple DNA repair diseases (Fig. 2A). Notably, we found three major disease clusters covering diverse phenotypes. The first cluster contains DNA repair diseases sharing neurodegeneration as the most prevalent feature (Fig. 2A, marked in red). Specifically, spinocerebellar ataxia with axonal neuropathy (SCAN1) and mutations in tyrosyl-DNA phosphodiesterase 1 (TDP1) gene; ataxia-telangiectasia-like disorder 1 with mutations in MRE11A gene; Cockayne syndrome carrying mutations in ERCC6 gene, and ten other conditions have been identified as diseases where neuronal function in brain is the primary target. Among these diseases, xeroderma pigmentosum has been shown to be associated with skin, oral and oropharyngeal cancers [31], while ataxia-telangiectasia patients displayed development of both solid tumors and more noticeable cancers of blood and lymphoid origin [15]. The second cluster corresponds to diseases with microcephaly, short stature and immunodeficiency as the main clinical phenotypes (Fig. 2A, marked in green). Increased frequency of cancer (both hematopoietic and solid malignancies) in this group was observed in patients with Nijmegen breakage syndrome caused by mutations in NBS1 [32]. The third group comprises of DNA repair disorders possessing considerable progeroid features, such as cardiovascular disease, and cancer prevalence (Fig. 2A, marked in purple). The type of malignancies across these clusters varies depending on gene mutation and syndrome. For instance, xeroderma pigmentosum groups B, E, F, G, V have been sub-clustered together and are known to be predisposed to skin cancer. Interestingly, Werner syndrome with mutations in the WRN gene has also been identified in the third cluster. Unlike other proteins involved in specific DNA repair mechanisms, WRN protein is known to play a critical role in regulating multiple DNA damage response pathways, in particular double-strand break repair. Werner syndrome patients develop sarcoma, skin cancers and other types of solid tumors. Notably despite the marked phenotypical differences both NBS1, ATM and WRN are critically involved in double-strand break repair. By comparing these three diseases we, therefore, hypothesize that we can discover pathways important for tumorigenesis while excluding other phenotypical characteristics. Hence, ataxia-telangiectasia, Nijmegen breakage syndrome and Werner syndrome have been selected for further analysis (Fig. 2A).

### CEP135 expression is perturbed in DNA repair diseases

To identify cancer-related pathways in these diseases, we analyzed changes in gene expression profiles using the PandaOmics platform. Comparison of transcriptomics signatures in disease and healthy samples revealed genes that are commonly either up- or downregulated in fibroblast cells derived from ataxia-telangiectasia, Nijmegen breakage syndrome and Werner syndrome patients (Fig. 2B). Genes associated with cell cycle progression and cytokinesis including CEP192, PAXIP1, CNTRL were identified as significantly perturbed among the top ten selected hits (Fig. 2C). Notably, CEP135 was the most downregulated gene with a similar pattern of expression across the three DNA repair diseases (Fig. 2D), suggesting that it may be associated with the shared cancer phenotype. Notably, CEP135 is involved in regulating centriole assembly and centrosome biogenesis [33]. Thus, downregulation of CEP135 together with other identified hits (Fig. 2C) may contribute to cell cycle dysregulation in DNA repair diseases [34–38]. On the other hand, alterations in this gene were also shown to be associated with centrosome amplification, which commonly occurs in cancer cells [39]. Based on these observations, quantification of CEP135 in individual cells has recently been proposed as a screening approach for tracking centrosome abnormalities during tumor progression [40]. We therefore hypothesized that CEP135 could be further used as a predictive biomarker to stratify patients into subgroups with different survival outcomes.

### CEP135 stratifies sarcoma patients with poor prognosis

**Fig. 3:**
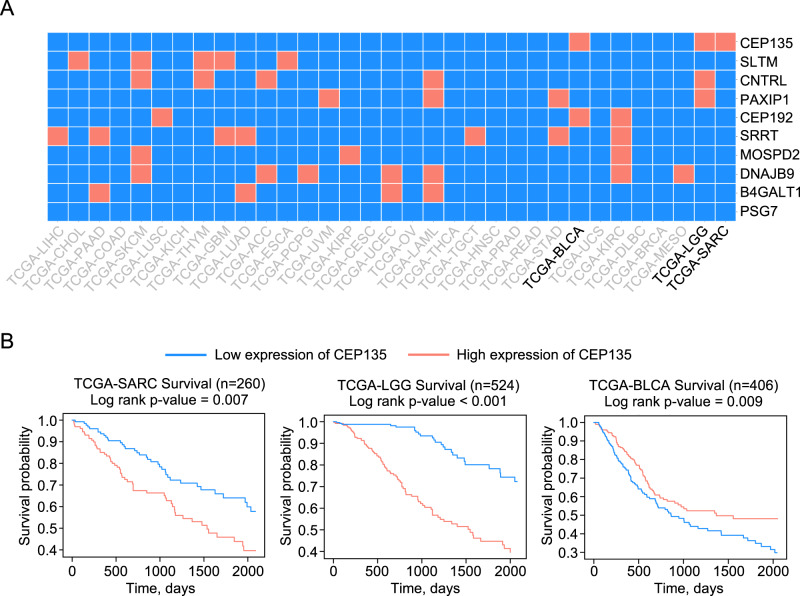
Survival analysis of cancer patients. **A** Survival analysis for top 10 genes among all available TCGA cancers. Results are presented on a heatmap and colored according to significance of the survival analysis. Red boxes correspond to significant differences in survival (*p*-value < 0.05, log-rank test) between groups of patients with high and low expression of analyzed gene, blue boxes denote non-significant changes. **B** Survival analysis for CEP135 is presented on a Kaplan–Meier plot for TCGA-SARC (Sarcoma), TCGA-LGG (Brain Lower Grade Glioma) and TCGA-BLCA (Bladder Urothelial Carcinoma) cancers.

To study whether CEP135 expression could be used as a biomarker capable of stratifying cancer patients with different outcomes, we performed survival analysis for thirty-three cancer types from TCGA dataset. Notably, nine of the ten genes that were found to be substantially perturbed in DNA repair diseases (Fig. 2C), were able to significantly stratify patients with at least three cancer types each based on their survival probability (Fig. 3A). Specifically, CEP135 was able to stratify patients with urothelial bladder carcinoma (BLCA), low-grade glioma (LGG), and sarcoma (SARC) cancers (Fig. 3A, B), with the most significant effect obtained in patients with LGG malignancies. Patients with high expression of CEP135 have a significantly lower survival compared to cases with low CEP135 levels (Fig. 3B). However, due to the difficulties in obtaining a tissue sample from brain cancers, CEP135 might be more suitable as a prognostic biomarker, rather than a predictive indicator of patients’ response to treatment. Interestingly, BLCA patients show an opposite trend, where high expression of CEP135 corresponds to better survival outcome. Notably, levels of CEP135 in sarcoma significantly defined patients with high and low survival outcomes based on the obtained *p*-value (Fig. 3B). CEP135 may, therefore, be potentially used as a predictive biomarker for sarcoma patients with poor survival, allowing the development of more tailored therapies for patients with unfavored clinical outcomes. As such, we next aimed to analyze the group of sarcoma patients with high expression of CEP135 and lower survival outcomes in the context of target discovery.

### PLK1 is a potential target in sarcoma patients

**Fig. 4:**
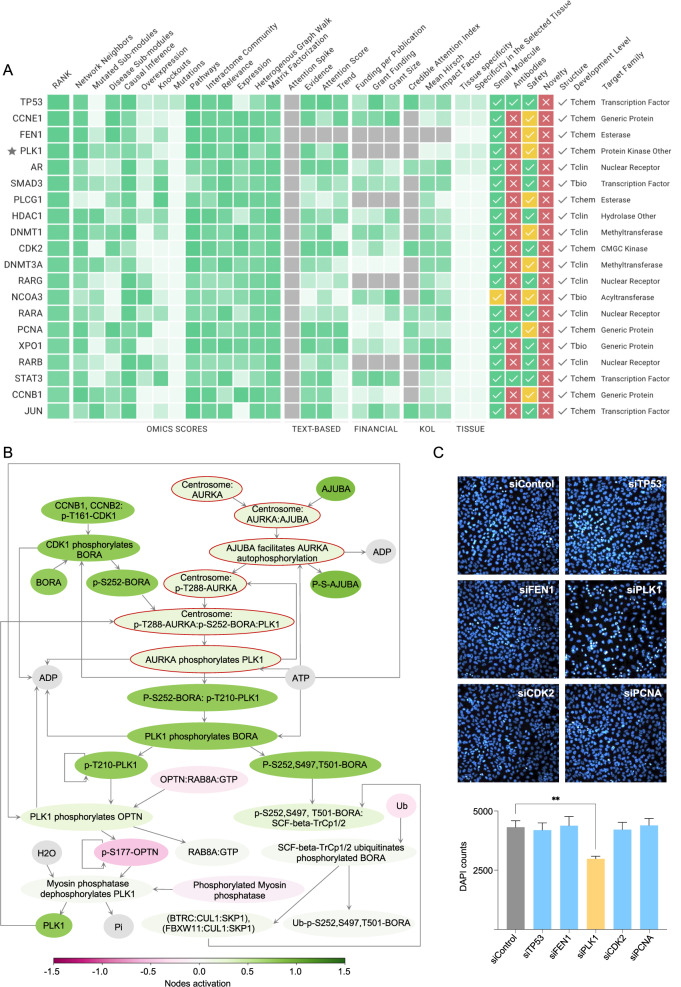
PandaOmics Target ID approach to identify targets in sarcoma patients and in vitro target validation. **A** Top 20 most promising targets for sarcoma patients with high expression of CEP135 vs adjacent normal samples derived from the In silico Target ID scoring approach. **B** Visualization of the pathway activation analysis for sarcoma patients with high CEP135 expression vs. low CEP135 expression for “Regulation of PLK1 Activity at G2-M Transition” (R-HSA-2565942) signaling pathway. All the nodes where CEP135 is encountered are highlighted with red circles. The coloring of the overall diagram corresponds to upregulation and downregulation of the particular nodes based on the gene expression changes and is colored as green and red respectively. **C** U2OS cells were incubated with corresponding siRNAs followed by staining with DAPI for nuclei quantification. Data are presented as absolute DAPI counts for three independent sets. The error bars show the error of mean. (**p-value < 0.01, non-parametric ttest).

For novel target identification we have applied Pandaomics-based Target ID algorithm to compare differences between the transcriptomic data derived from sarcoma patients with low survival and high expression of CEP135 and non-tumorous tissue samples. Among the list of top twenty predicted targets, several known genes were identified that play a role in cancer cell survival and apoptosis including TP53, CCNE1, FEN1, STAT3 and others (Fig. 4A). Notably, polo-like kinase 1 (PLK1), one of the crucial enzymes associated with cell cycle division and cytokinesis, has been identified as one of the top hits (Fig. 4A). Importantly, similar results have been obtained when comparison between two groups of sarcoma patients with high and low expression of CEP135 has been performed (data not shown). These groups have further been used to investigate differences on the pathway activation level (Fig. 4B) using the iPANDA algorithm [41] A specific signaling pathway named “Regulation of PLK1 Activity at G2-M Transition” (R-HSA-2565942) was among the 5% of most significantly upregulated pathways in sarcoma patients with poor health outcome (Fig. 4B). Notably, according to the pathway network topology, CEP135 is an important upstream regulator of PLK1-mediated phosphorylation of the key players of G2-M checkpoint (Fig. 4B, nodes circled in red). Activation of PLK1 and upregulation of the PLK1-mediated signaling pathway in sarcoma patients could, therefore, be due to the high expression of CEP135, an associated upstream PLK1 regulator. Experimental verification was further performed by siRNA-based genetic silencing of identified targets in human osteosarcoma U2Os cells (Fig. 4C). In total, five genes have been selected (TP53, FEN1, PLK1, CDK2, and PCNA) and cell nuclei have been quantified 48 h after transfection. Out of five targets, only knockdown of PLK1 showed significant reduction in cell growth (Fig. 4C), posing PLK1 as a promising target for a subset of sarcoma patients with high CEP135 expression and poor survival rates.

## Discussion

It takes over 10 years to bring a drug to the market, the process costs close to $2 Billion US dollars and fails over 90% of the time. Most of the failures transpire in early preclinical research where targets are discovered and tested in experimental models and in Phase II clinical studies in humans where efficacy of the drug is tested in humans primarily testing the disease hypothesis, target choice, biomarker selection, and clinical trial design. On average over 66% of the Phase II studies fail. Target discovery is the most important and critical step in drug discovery and development [42]. Hence, target discovery and target choice for preclinical research is usually performed by the human experts relying on very different philosophies. These philosophies may include evidence from multiple animal studies published in high-profile peer-reviewed journals, targets found using large amounts of clinical data, genetic mutation-driven targets, gene or protein expression-derived targets and many other approaches. There is no single correct method to identify a target and the balance between novelty and confidence. Here, we decided to utilize the PandaOmics platform, which integrates multiple target discovery approaches based on different data types including text and multiomics, and implements multiple AI and bioinformatics algorithms.

The approach utilized in the current study demonstrates how AI-powered PandaOmics analysis of gene expression signatures in cancer-associated diseases could lead to identification of novel biomarkers for specific cancer patient subgroups and detection of related therapeutic targets. The efficiency of the PandaOmics platform has recently been shown for prediction of novel age-associated targets for drug discovery purposes [43]. Importantly, its capabilities for target discovery cover both analysis across multiple types of diseases as well as in depth characterization of key molecular players driving the progression of a specific disease [44]. This includes, for instance, the identification of cyclin-dependent kinase 20 (CDK20) as a novel target in hepatocellular carcinoma and generation of potent CDK20 inhibitors using the generative chemistry platform Chemistry42 coupled with subsequent in vitro explorations [30, 45]. Another example refers to the prompt discovery of potent inhibitors against discoidin domain receptor 1 (DDR1) kinase including *de novo* small molecule design, synthesis and in vivo validation [46]. Analysis of multiomics-based data and application of PandaOmics has been further highlighted for acceleration of advancements in treatment of Duchenne muscular dystrophy [47]. In this regard, the possibilities for AI-driven target discovery became broadly unleashed for remarkably faster progression of discovered hits into clinical trials. This particularly includes combining datasets derived from various platforms and step-by-step characterization and categorization of predicted targets in the context of disease, novelty and druggability. Such comprehensive integration of multiple data sources as well as the analyses of various outputs for the specific research task confers PandaOmics platform a number of advantages, compared to the other online platforms for biomarker and target discovery, such as ToPP [48]. Similar to PandaOmics, ToPP may perform mutliomics-based examination of the various data sources including but not limited to transcriptome, proteome and epigenome for tumor prognostic studies. Furthermore, ToPP may also be used for the biomarker-based survival analyses across TCGA datasets applying either publicly available or private data with the possibility of input customization and output parameters including charts, thresholds, patients selection criteria, etc. While ToPP and other available platforms [QIAGEN IPA, https://www.qiagen.com/), MetaCore (https://clarivate.com/metacore), Elsevier’s Pathway Studio (https://www.pathwaystudio.com/) and Genevestigator (https://genevestigator.com/)] greatly expands the ability to perform big data analyses, PandaOmics provides a broader scale of analytical capabilities, due to integration of the pathway interpretation and activity analysis, target drugability report, using grant and funding data sources and AI application. One of the unique features of PandaOmics target discovery platform is the validation pipeline used to estimate the performance of the models. To this end, we developed a Time Machine approach, where we trained the computational models using data published before a certain time point and validated the model outputs by their ability to predict those targets that came into the focus of the pharmaceutical industry after this time point. The power of PandaOmics has, therefore, been redirected in our case into target discovery in cancer research due to the absence of appropriate biomarkers, specific molecular targets driving tumor progression and limitations of existing treatments for multiple cancer types with different grades [49–52].

Specifically, an initial step in our current work included an analysis of DNA repair disorders because of the known tight connection between cancer development and genome instability. Particularly, the DNA damage response is impaired in multiple cancers with the downregulation of some pathways dictating sensitivity of cancer cells to DNA-damaging agents while others are upregulated bestowing resistance to chemo- and radiation therapy [53]. Notably, we selected distinct DNA repair disorders where cancer is a shared clinical phenotype using hierarchical clustering, a correlation analysis that has successfully been applied before for identification of co-associated aging features [54]. Discovery of the top genes that are significantly perturbed among cancer-predisposed DNA repair diseases led to our further investigation of their significance as marker genes considering that genetic alterations driving cancer progression vary between individual patients. Since different patients with the same cancer diagnosis show diverse sensitivity patterns for selected therapy, biomarker-based stratification would, therefore, allow patients to benefit the most from advanced tailored therapies [55]. CEP135 was identified as the most significantly perturbed gene among the studied DNA repair disorders and its expression correlates with the severity of survival of sarcoma patients where high expression of CEP135 is associated with poor survival probability. Notably, observed in our settings downregulation of CEP135 in DNA repair diseases (Fig. 2D) is likely to be related to the cell cycle alterations that occur in premature aging, while its elevated expression in cancer goes in line with the known function of CEP135 in centrosome biogenesis and associated cancer-related abnormalities, including centrosome amplification and subsequent tumor progression [39]. Interestingly, such dual roles have been shown for other genes including the well-characterized function of transforming growth factor-beta (TGFbeta), which may act as both a tumor suppressor and oncogene [56, 57]. Collectively, these results highlight the importance of considering CEP135 as a novel marker gene for further investigations, since its association with sarcoma has not been well-characterized. Besides CEP135, current work provides data for the rest of the top ten genes that significantly correlate with survival probability among thirty-three cancer types and could be used by researchers as indications for novel biomarker discovery and verification.

Importantly, identification of novel targets specifically associated with a low probability of survival in sarcoma patients, and which may act as drivers of tumor progression, is of particular interest. PandaOmics-based TargetID revealed hits that may potentially be considered as putative targets for sarcoma patients. Among them, PLK1 was selected, and in vitro experiments verified its contribution to cell survival. Since PLK1 possesses a key role for centriole duplication and G2/M transition in cell cycle [58, 59], identification of this kinase in the same signaling cascade together with CEP135, pathway that was shown by us to be activated in subgroup of sarcoma with high CEP135 expression, supports PLK1 as a plausible target candidate. Recently, PLK1 was proposed as a target for the treatment of the five solid tumor types including soft tissue sarcoma, where application of potent PLK1 inhibitor showed limited antitumor activity in recruited patients [60]. Presumably poor recruitment strategy was the reason the trial did not reach its efficacy endpoints. It highlights the significance of the usage of molecular biomarkers to improve patients’ selection criteria. In this regard, CEP135 could be proposed as a novel predictive biomarker for sarcoma patient stratification.

In conclusion, our study provides evidence of the possibility of developing advanced tailored therapies to improve the outcomes of sarcoma patients. The latter is based on patient stratification through appropriate marker genes. In this regard, the application of in silico-based approaches such as PandaOmics may accelerate this step and subsequent target identification not only for cancer but also for a broader range of age-associated diseases.

## Materials and methods

### Hierarchical clustering

Clustering of diseases based on phenotypes was done as previously described [61]. The prevalence of features was retrieved from published datasets for each disease and were used as input vectors for agglomerative hierarchical clustering using uncentered similarity metrics and average linkage (www.mitodb.com).

### Data collection

Gene expression data were collected from publicly available repository Gene Expression Omnibus (GEO) [62]. Transcriptomics datasets obtained from fibroblasts and/or induced pluripotent stem cells (iPSCs) included GSE48761 (10 samples from healthy patients and 10 samples from patients with Werner syndrome) [63]; GSE94707 (7 samples from healthy patients and 4 samples from patients with Nijmegen Breakage syndrome) [64]; GSE35347 (6 samples from healthy patients and 3 samples from patients with Ataxia-Telangiectasia syndrome [65]; GSE152287 (5 samples from healthy patients and 5 samples from patients with Ataxia-Telangiectasia syndrome) [66]. TCGA GDC HTSeq – FPKM-UQ expression data from tumor and adjacent non-tumor tissues were collected from UCSC Xena database [67]. Gene expression data were uploaded into PandaOmics (https://insilico.com/pandaomics) and pre-processed according to PandaOmics pipeline that automatically defines data type (raw counts, normalized gene expression values or log-transformed gene expression values) and recommends normalization method for further analysis. Upper-quartile normalization and log2-transformation were applied for datasets derived from the GEO database. For cancer datasets derived from UCSC Xena database only log2-transformation was applied as data were already normalized.

### Differential expression analysis

Differential analysis has been performed using the *limma* package for microarray data. Each dataset has been processed according to standard protocols. Obtained gene-wise *p*-values were corrected by Benjamini–Hochberg procedure. Common gene expression signatures among Ataxia-Telangiectasia syndrome, Nijmegen Breakage syndrome and Werner syndrome were analyzed in the meta-analysis section of PandaOmics. Meta-analysis section allows to calculate combined logarithmic fold-changes (LFC) and *Q*-values across all used for the analysis gene expression datasets using *minmax* normalization for LFC values and stouffer’s method combining *p*-values with further FDR correction. Accordingly, heatmaps and a volcano plot have been created for top 10 most significant genes that are perturbed among all three diseases. Gene expression changes between disease and control groups were plotted on a box-plot and two-tailed Mann–Whitney test was used to calculate the statistical significance.

### Overall survival analysis and stratification of cancer patients

Survival analysis was prepared in PandaOmics using the *KaplanMeierFitter* function from *lifelines* python package. Median function was applied for normalized gene expression data and median value for each gene of interest was used as a threshold for patients’ stratification. Patients with the expression value of the gene of interest >, =, or < than median value were considered as patients with “high” or “low” expression of a particular gene, respectively. Log-rank test was used to calculate the statistical significance. Briefly, 33 TCGA GDC cancers were applied, and survival analysis was performed for the top 10 significantly perturbed genes identified from differential analysis. Probability of survival was plotted on a heatmap and colored as red if there was a significant difference between high and low expression of the gene and blue if there was no significant difference. Individual survival plots for CEP135 gene were plotted using *matplotlib* package.

### Pandomics TargetID platform for target identification

In silico-based PandaOmics target discovery/scoring approach was applied to identify novel molecular targets for stratified sarcoma patients. This approach is based on the combination of multiple scores derived from text and omics data. Text-based scores are derived from various sources including scientific publications, grants, patents, clinical trials and the key opinion leaders, and thus represent how strongly a particular target is associated with a disease. Specifically, text-based scores contain Attention, Trend, Attention Spike, Evidence, Grant funding, Funding per Publication, Grant Size, Average Hirsch, Impact Factor and Credibility attention index scores. For example, Attention score reflects the total number of the gene of interest, which was mentioned in the text data described above across all time periods (as both disease-agnostic and disease-specific). In contrast, omics scores are based on the differential expression, GWAS studies, somatic and germline mutations, interactome topology, signaling pathway perturbation analysis algorithms, knockout/overexpression experiments and more omics-data sources, and thus represent the target-diseases association according to molecular connections between proposed target and disease of interest. Omics scores include thirteen models (Heterogeneous Graph Walk, Matrix Factorization, Interactome Community, Causal inference, Overexpression/Knockout, Mutated/Disease Sub-modules, Mutations, Pathway, Network Neighbors, Relevance and Expression) that can be subdivided into classic bioinformatics approaches and complex AI-based models. For example, the Expression score relies on the combination of each gene’s fold change difference in disease versus control samples, the statistical significance of this change, and basal expression in the disease-relevant tissue. On the other hand, AI-based omics model called Heterogeneous Graph Walk (HeroWalk) is a guided random walk-based approach that is applied to a heterogeneous graph. The model learns node representations and then identifies gene nodes, which are close to the reference disease node. The “walks” are sampled with a predefined meta-path, i.e., fixed sequence of node types in a walk, e.g., “gene”–“disease”–“gene”. The node degree controls the probability of transition between the nodes while sampling, following by the SkipGram model [68] that learns the representation of each node based on the resulting corpus of walks. The cosine similarity between the specific disease and all genes produces a ranked list of genes. The top genes from this list are predicted to be promising target hypotheses. All the models regardless of the particular methodology output the ranked lists of target hypotheses. Combination of described scores results in a ranked list of targets proposed for a given disease and can be filtered out in regard to their novelty, small molecules synthesis availability, availability of PDB structure and other useful filters. Description of all mentioned scores and filters is available in the User manual section of PandaOmics (https://insilico.com/pandaomics/help). For the current study, target ID scoring was calculated for the comparison between gene expression data for stratified sarcoma patients with low survival prognosis and high expression of CEP135, with adjacent non-involved tissue samples free of atypical cells. To identify novel molecular targets for stratified sarcoma patients, only omics-based scores were taken into account and used for the composition of the scores for ranking.Targets that are not druggable by the small molecules and do have red flags in terms of safety were excluded from the analysis. In other words, the aim of the target ID scoring was to identify potential target hypotheses for stratified groups of cancer patients. After filtering, a ranked list of proposed targets was extracted from PandaOmics and presented as a heatmap.

### Pathway activation analysis

Analysis of perturbed pathways was performed in PandaOmics using iPANDA algorithm [41] for comparison between sarcoma patients with high CEP135 expression vs sarcoma patients with low CEP135 expression from TCGA-SARC dataset. Reactome signaling pathway graph was used as a database for iPANDA algorithm [69]. This method estimates direction and intensity of a pathway activation using a linear combination of logarithmic fold-changes, statistical and topological weights applied for each pathway member gene and described in [41]. As a result, iPANDA’s algorithm output represents how changes in gene expression between case and control groups affect signaling pathway levels and score for each pathway is defined as iPANDA score. High values of iPANDA scores correspond to the upregulation of the pathways, while low values correspond to the downregulation. Pathway perturbation is represented on a pathway scheme and each node is colored based on the average LFC values across the genes in the node.

### Cell culture

Human osteosarcoma (U2Os) cell line was purchased from ATCC (HTB96TM). Cells were cultured at 37 °C and 5% CO_2_ in 4.5 g/L glucose DMEM-GlutaMAX media (Gibco, 12077549) supplemented with 10% fetal bovine serum (Sigma-Aldrich, F9665) and 100 U/mL penicillin–streptomycin (Gibco, 15140163).

### siRNA-mediated knockdown of selected hits

Transient knockdown of selected genes of interest with small-interfering RNAs (siRNA) was performed using Lipofectamine™ RNAiMAX Transfection Reagent (Invitrogen, 13778150). The following siRNAs were used: silencer select PLK1 (Thermo Scientific, 4390824), silencer select negative controls (Thermo Scientific, 4390846, 4390843). TP53, CHK2, PCNA and FEN1 siRNAs were part of Silencer™ Select Human DNA Damage Response siRNA Library (Invitrogen, A30089). Briefly, 10 nM siRNAs were incubated with RNAiMAX-OptiMEM (Gibco, 51985026) mixture for 15 min at RT in the 384-well plate (Greiner bio-one, 781091). Confluent U2Os cells were washed twice with warm phosphate-buffered saline (PBS), trypsinized and added to the transfection solution in a density of 2000 cells per well. After 48 h incubation, cells were washed with warm PBS and fixed in 4% PFA (Santa-Cruz, sc-281692) for 15 min. Cells were washed three times with PBS followed by permeabilization in 0.1% triton X100-PBS for another 10 min. Cells were washed again three times with PBS and stained with DAPI (PanReac AppliChem, A4099) for 10 min. Cells were washed ones with PBS and stored at +4 °C until the analysis. DAPI nuclei were imaged using Incell analyzer 2200 high-content microscopy at ×20 magnification.

## Data Availability

All data supporting the conclusions of the paper are available in the article and corresponding figures. GEO and TCGA datasets used in the paper are described in materials and methods section.
